# Znet: Deep Learning Approach for 2D MRI Brain Tumor Segmentation

**DOI:** 10.1109/JTEHM.2022.3176737

**Published:** 2022-05-23

**Authors:** Mohammad Ashraf Ottom, Hanif Abdul Rahman, Ivo D. Dinov

**Affiliations:** Department of Information SystemsYarmouk University 59179 Irbid 21163 Jordan; Statistics Online Computational ResourceDepartments of Health Behavior and Biological Sciences and Computational Medicine and BioinformaticsUniversity of Michigan 1259 Ann Arbor MI 48109 USA; PAPRSB Institute of Health Sciences, Universiti Brunei Darussalam 37597 Gadong BE1410 Brunei Darussalam

**Keywords:** Brain tumor, region segmentation, deep learning, augmentation, neural networks

## Abstract

Background: Detection and segmentation of brain tumors using MR images are challenging and valuable tasks in the medical field. Early diagnosing and localizing of brain tumors can save lives and provide timely options for physicians to select efficient treatment plans. Deep learning approaches have attracted researchers in medical imaging due to their capacity, performance, and potential to assist in accurate diagnosis, prognosis, and medical treatment technologies. Methods and procedures: This paper presents a novel framework for segmenting 2D brain tumors in MR images using deep neural networks (DNN) and utilizing data augmentation strategies. The proposed approach (Znet) is based on the idea of skip-connection, encoder-decoder architectures, and data amplification to propagate the intrinsic affinities of a relatively smaller number of expert delineated tumors, e.g., hundreds of patients of the low-grade glioma (LGG), to many thousands of synthetic cases. Results: Our experimental results showed high values of the mean dice similarity coefficient (dice = 0.96 during model training and dice = 0.92 for the independent testing dataset). Other evaluation measures were also relatively high, e.g., pixel accuracy = 0.996, F1 score = 0.81, and Matthews Correlation Coefficient, MCC = 0.81. The results and visualization of the DNN-derived tumor masks in the testing dataset showcase the ZNet model’s capability to localize and auto-segment brain tumors in MR images. This approach can further be generalized to 3D brain volumes, other pathologies, and a wide range of image modalities. Conclusion: We can confirm the ability of deep learning methods and the proposed Znet framework to detect and segment tumors in MR images. Furthermore, pixel accuracy evaluation may not be a suitable evaluation measure for semantic segmentation in case of class imbalance in MR images segmentation. This is because the dominant class in ground truth images is the background. Therefore, a high value of pixel accuracy can be misleading in some computer vision applications. On the other hand, alternative evaluation metrics, such as dice and IoU (Intersection over Union), are more factual for semantic segmentation. Clinical impact: Artificial intelligence (AI) applications in medicine are advancing swiftly, however, there is a lack of deployed techniques in clinical practice. This research demonstrates a practical example of AI applications in medical imaging, which can be deployed as a tool for auto-segmentation of tumors in MR images.

## I. Introduction

Recent advancements in brain imaging, information technologies, and digital health records have opened the door for enormous progress in holistic examination and deep phenotyping of the human brain. Structural, functional, diffusion, and spectroscopy explorations are now possible using Magnetic Resonance Imaging (MRI) [1], [2]. These advances are enabling health practitioners, radiologists, and medical scholars to peer into the anatomical and physiological organization of the brain, accurately diagnose normal and pathological development, maturation, and aging, as well as assist health professionals and clinicians. Manual inspection, segmentation, and analysis of MR images can be challenging for a number of reasons. The processing of large volumes of complex multiresolution data, including series of images, volumes, and participants; requires significant time, effort, and unique expert skills, e.g., long training process and collaborating teams of radiologists, neurologists, and data scientists. Detection of normal and pathological traits is subjective and includes a significant margin of error [1], [3]. Computational and Artificial Intelligence (AI) techniques [4]–[6] can significantly assist with building interpretable models, trained on existing medical images to automatically identify patterns, derive labels, and diagnose new cases [7]–[9]. In general, AI models are not intended to replace, but rather to complement, assist and enhance human medical expertise. Derived computational models can optimize and expedite scientific research and clinical practice. Medical image segmentation is the process of recognizing, detecting, and labeling anatomical parts, physiologically relevant areas, or the intrinsic network organization of the brain. Segmentation brain maps facilitate clinical decision-making and provide a core step in computer-aided diagnostics. The literature categorizes segmentation into three types: automatic, semi-automatic, and manual segmentation. Generally, there are three main models to localize brain region boundaries as curves in 2D images or surfaces in 3D volumes, e.g., brain tumor regions [10], [11]. These include traditional machine learning approaches, atlas registration, and deep learning methods. In classical machine learning image segmentation, expert-derived manual segmentation masks are employed to train a machine learning classifier. Label annotation tools are used to identify the target features, objects, or shapes. Machine learning algorithms are used for training and then for testing the model to detect analogous shapes and features in prospective out-of-bag (new) datasets. For instance, a statistical learning approach was used to model brain anatomical structure segmentation and develop a probabilistic brain atlas [12], [13]. Another example, OpenLabeling (python tool) is used to label tumors in medical images, and a support vector machine is used to produce the model. This approach is considered time-consuming and requires domain experts to obtain the initial manual annotations. In addition, the performance is relatively poor when the border between malignant and benign tissues is ambiguous [14].

Multi-Atlas segmentation (MAS) is a semi-automatic segmentation method that relies on prior atlases information. MAS was evolved from the single-atlas method but is considered insufficient for medical segmentation due to the lack of prior information, which leads to low performance and suboptimal accuracy [15]. As an alternative, multi-atlases with more prior information are used for segmenting novel images. For example, employing pairwise registration between each atlas image and the novel image, where the registration results are used to propagate the atlas labels into the space of the new image coordinates, which is followed by the selection of the most likely labels for each voxel [16].

**FIGURE 1.**
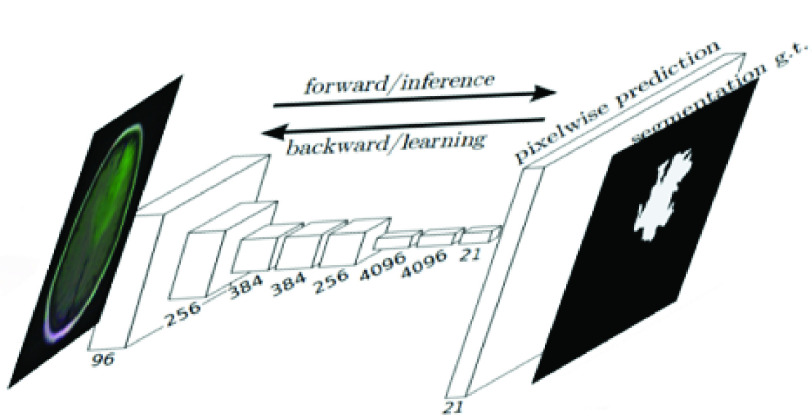
Fully convolutional network [18].

In recent years, deep learning methods for medical image segmentation have received lots of attention, due to multiple reports with outstanding results for finding and predicting the target shapes in the images. The literature includes many algorithms for segmentation, but the fully convolutional network (FCN) and convolutional autoencoder methods are considered the most effective methods for medical image segmentation [17]. A fully conventional network (FCN) consists of a set of layers where each layer outputs is a three-dimensional array of size $hxwxc$, where $h$ and $w$ are height and width respectively, and $c$ is the channel number. The input image is considered as the first layer, and the subsequent layers are the output of convolving previous layers, the basic elements of each layer are convolution, pooling, and activation functions. Writing $x_{i,j}$ for the data vector at location $(i, j)$ in a particular layer, and $y_{i,j}$for the following layer, these functions compute outputs $y_{i,j}$ by the following equation (eq. 1) [18]:

\begin{equation*} y_{i,j}=f_{k,s}\left ({\{x_{s_{i}+\delta _{i}, s_{j}+\delta _{j}} \}~| ~0\leq \delta _{i}, \delta _{j} \leq k }\right)\tag{1}\end{equation*}

where $s$ denotes the stride, $k$ is the kernel size, and $f_{k,s}$ determines the layer type. The final output layer in (FCN) will be the same size as the input layer (input image), However, the number of channels is determined by the classification classes number where one channel means one class which is normally used to obtain the predicted mask (desired output). Figure 1 shows the framework for the fully convolutional networks for medical image segmentation.

Autoencoder methods were utilized to extract features from images or inputs sample while attempting to keep most of the original information. The autoencoder consists of two main parts, the encoder and decoder. The encoder part is to encode the input images into smaller dimensional intermediate representations, where the decoder is to reconstruct the original input images from the intermediate representation. The loss function in autoencoders is calculated in terms of the similarity between input images and the reconstructed images [19], [20].

**FIGURE 2.**
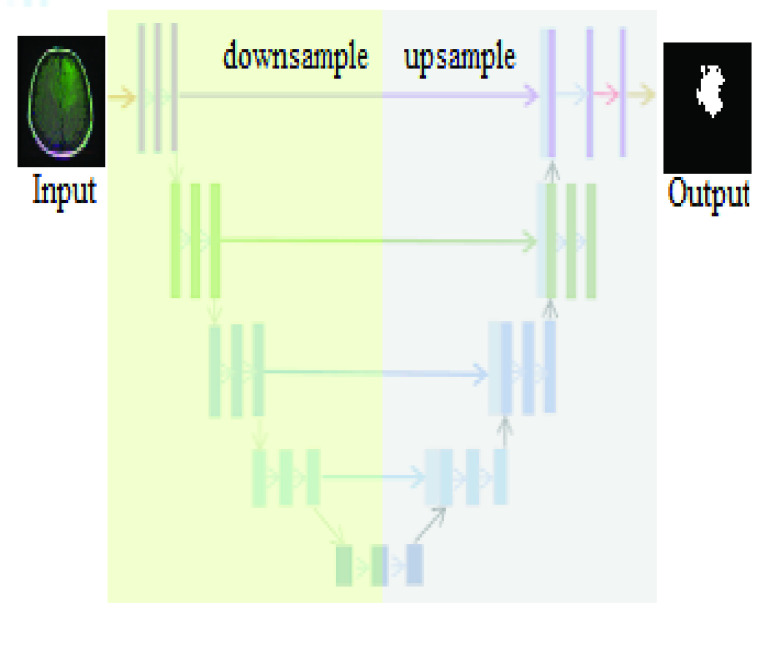
Unet architecture [22], an extension and modification of fully convolutional network, receives a new MR image and produces the mask tracking the shape of the detected tumor.

Unet is an extension of fully convolutional network architecture to work with a limited number of training data and to produce more accurate segmentation [21], Figure 2 shows the Unet architecture, which consists of a contracting path (left side) and an expansive path (right side). The left side of the network represents the normal architecture of a convolutional network, and each block contains two $3\times 3$ convolutions, rectified linear unit (ReLU), and a $2\times 2$ max pooling respectively; this process is called down-sampling. At the end of each contracting block, the image size is reduced, and feature channels are doubled. The right side of Unet architecture is called expansive path, which consists of an up-sampling of the feature map followed by a $2\times 2$ convolution that reduces the number of feature channels by half. Unet also applies concatenation between the output of each block on the left side of the diagram with the corresponding cropped block on the right side, cropping or matching arrays sizes are essential to combine arrays into one array, and then apply another two $3\times 3$ convolutions followed by a ReLU activation function. At the final block, a $1\times 1$ convolution is used to produce the output [22]. A key feature in the Unet architecture is the use of a large number of feature channels, and the use of skip connections. The purpose of skip connections in convolutional networks is to enhance gradient flow through a large number of layers to focus on more features in the image and to generate shapely segmentation [23].

In the current literature, some studies on MRI tumor segmentation use alternative methods, such as convolutional networks, random forests, and Unet. A hybrid approach of Unet and RESNET (residual networks) has been proposed by [24], [25] yields high performance on brain tumor segmentation, dice = 0.90. Ankari *et al.* [26] introduced genetic algorithms (GA) with convolutional networks to perform brain tumors segmentation. GA aims to obtain the optimal CNN structure rather than the standard trial way of building the convolutional network. This approach constructs several networks with the highest accuracy of 0.94 on the TCGA dataset. Several experiments were performed by [27] for brain tumor segmentation using Feature Pyramid Networks (FPNs), RESNET, and Unet on TCGA (The Cancer Genome Atlas [28]) dataset. The reported validation results show optimal performance with dice = 0.93 by using RESNET. A recent study [14] intended to build an effective segmentation system for brain tumors using deep learning methods, specifically Cascade Convolutional Neural Network (C-CNN). Their approach depends on a small amount of data rather than using the entire dataset, which overcomes high computation time constraints, restricts resource use, and reduces the overfitting problem. This method also reported competitive results with dice = 0.92. Another study [29]] modified the Unet framework by replacing the deconvolution block with the Nearest Neighbor method and two convolutional layers. It achieved promising results for segmenting brain tumors with dice = 0.88 and IoU = 0.86. Goetz *et al.* [30] used the ExtraTrees algorithm to segment brain tumors. Since ExtraTrees provides more randomness during the training phase and their reported dice = 0.83.

Automated clinical diagnosis, detection of clinical phenotypes, and forecasting of biomedical attributes and health phenotypes can be subjective and error-prone. AI methods can augment human experts by deriving unbiased, reliable (low variability) and quick computer models that can assist medical professionals with identifying patterns (such as tumors) and suggesting optimal interventions. The applications of AI in medicine are advancing swiftly. Yet, there is caution to expeditious, blind, and ubiquitous immersion of AI in all aspects of clinical practice. This research demonstrates one pragmatic case where the application of AI in medical imaging provides a robust estimation of the presence and dynamics of brain tumor development. Following broad and deep performance assessment, such AI applications can be deployed as desktop tools or mobile apps for automated detection, classification, and tracking of brain tumors using MR images.

## II. Methodology

### A. Dataset

The dataset used in this research study is a public benchmark dataset based on The Cancer Genome Atlas Low-Grade Glioma (TCGA - LGG) [31], [32]. The idea behind TCGA – LGG compilation is to build cancer images for research purposes and to study the relationship between phenotype to genotype in cancer and medical images research. The dataset was obtained during treatment and follow-up at multiple locations, as shown in Table 1 [31].

**TABLE 1:** Institutions and Individuals Contributed to TCGA-LGG Data Collection

Institution Name	Number of patients
Henry Ford Hospital, Detroit	46 patients
ThomasJefferson University, Philadelphia	16 patients
Case Western Reserve University, Cleveland	48 patients

**FIGURE 3.**
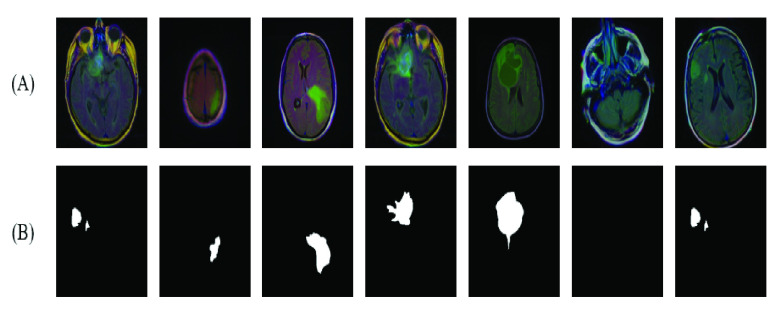
(A) Samples of dataset images (B) the corresponding annotated ground truth (tumor mask).

Budaet *et al.* [32] investigated the dataset in [31] and made it available for computational research and medical image processing. In addition, our focus is on the FLAIR MRI images, which contain LGG enhanced tumor images. Neuroradiology experts manually reviewed, annotated, and labeled the FLAIR images for 110 patients. For pathological cases, the experts delineated the shape boundary of the FLAIR abnormality (tumor) for each image. The original MRI and the manually labeled masks represented the training dataset. The total number of image slices obtained was 3,929, with 2,556 labeled as normal and 1,373 abnormal (tumor). A few random samples of the dataset are shown in Figure 3.

### B. Data Preprocessing

Randomly, the dataset was divided into training (3005 images), validation (393 images), and testing (432 images). The validation represents about 10% of the original dataset, testing images represent about 15% of the training dataset, and the remaining images are for training the model. Images resolution in the original dataset was $256\times 256$, and due to extensive processing and computation time for the size of the original image, we resized data to $128\times 128$ pixels.

**FIGURE 4.**
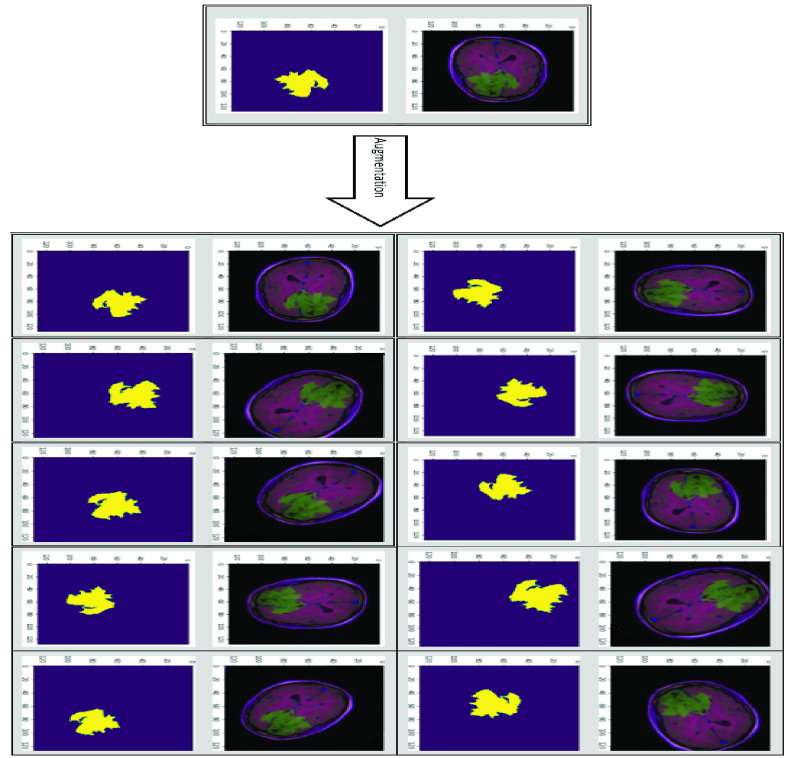
Samples of data augmentation using albumentations python library techniques.

Imaging data augmentation became a popular pre-processing step in data science and AI applications, it is mainly used when training data is limited or when the amount of data is important to produce a better computer model, in addition, data augmentation can reduce the overfitting problem and increase model performance [33]. In this research, we used Albumentations, a free and open-source python library to perform the necessary images augmentation [34]. Albumentations processes provided a broad and holistic data augmentation. HorizontalFlip was used to produce new images from the existing images based on flipping the input around the y-axis of input horizontally. Albumentations VerticalFlip was utilized to generate new images by flipping the input around the x-axis vertically. RandomRotate90 was employed to rotate the input Randomly by 90 degrees. Transpose was applied to exchange rows and columns in the input image. Random affine transformations using ShiftScaleRotate were applied to generate new images based on shifting, scaling, and rotating the input images. Finally, all input images pixels were converted to floating-point elements in the range between 0 and 1. Figure 4 shows sample data augmented by Albumentations python library techniques.

## III. Znet Framework

**FIGURE 5.**
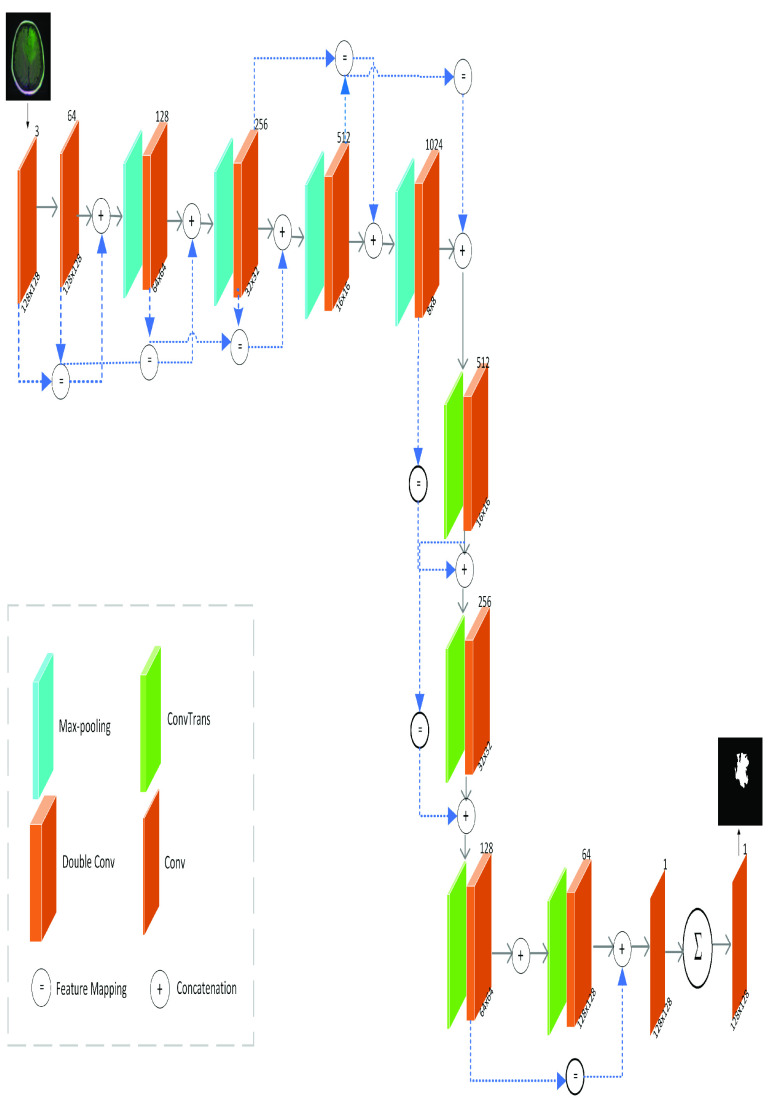
Proposed architecture of the Znet.

In the proposed network architecture, we utilized the concepts of two well-known segmentation methods to construct a novel framework for MR images tumor segmentation; adversarial networks (AN) and Unet method. The framework uses the principle of skip connections and concatenating tensors from the AN and encoder-decoder design from the Unet method. Figure 5 shows the complete architecture of the proposed Znet method. The architecture consists of an encoding part (analytic downsampling) and a decoder part (synthetic upsampling).

The encoder part consists of five blocks, where each encoder block contains double convolutions combined by batch normalization and rectified activation function ReLU (equation 2) and followed by max-pooling. The output of the encoder block is concatenated with the encoder block input. Note, the encoder block input is interpolated to match the feature map of the encoder block output.

\begin{align*} ReLu(z)=&\begin{cases} \displaystyle 0, & z \leq 0\\ \displaystyle z, & otherwise \end{cases} \tag{2}\\ \sum (z)=&\frac {1} {1 + e^{-z}} \tag{3}\\ l_{n}=&- w_{n} \left [{ y_{n} \cdot \log x_{n} + (1 - y_{n}) \cdot \log (1 - x_{n}) }\right]\tag{4}\end{align*}

where $w$ is the optional weight, $y$ and $x$ are the target and input respectively. The decoder part consists of five blocks, the same as the encoder block, except for the use of ConvTranspose2d instead of max-pooling.ConvTranspose2d is usually used to enlarge a tensor at the end of each decoder block, so we finally get the original image dimensions in the last decoder block. The final block is the output block which consists of a single convolution and sigmoid activation function (equation 3). We trained the model for 200 epochs using adaptive moment estimation (ADAM) optimizer [35] (equation 4),three channels $128\times 128$ pixels, a batch size of 32, and binary cross-entropy loss function [36]. Table 2 shows hyper-parameters used for training the proposed model, and Table 3 shows a sample of the architecture summary.

**TABLE 2:** Hyper-Parameters Used for Training the Proposed Model

Parameter	Name
Epoch	200
Optimizer	Adam
Batch Size	32
Learning Rate	$lr=5*e*-4$
Input Size	$3\times 128\times 128$

**TABLE 3:** The Proposed Method Architecture Summary

Layer (type)	Output Shape	Param #
Conv2d	[−1, 64, 128, 128]	1,792
BatchNorm2d	[−1, 64, 128, 128]	128
ReLU	[−1, 64, 128, 128]	0
Conv2d	[−1, 64, 128, 128]	36,928
BatchNorm2d	[−1, 64, 128, 128]	128
ReLU	[−1, 64, 128, 128]	0
:	:	:
:	:	:
ConvTranspose2d	[−1, 128, 128, 128]	65,664
Conv2d	[−1, 64, 128, 128]	73,792
BatchNorm2d	[−1, 64, 128, 128]	128
ReLU	[−1, 64, 128, 128]	0
Conv2d	[−1, 64, 128, 128]	36,928
BatchNorm2d	[−1, 64, 128, 128]	128
ReLU	[−1, 64, 128, 128]	0
Conv2d	[−1, 64, 128, 128]	12,352
BatchNorm2d	[−1, 64, 128, 128]	128
ReLU	[−1, 64, 128, 128]	0
OneConv	[−1, 64, 128, 128]	0
Conv2d	[−1, 1, 128, 128]	65
Sigmoid	[−1, 1, 128, 128]	0
OutConv	[−1, 1, 128, 128]	0

### A. Evaluation Metrics

The most common metrics for evaluating the performance of medical MR images segmentation include pixel accuracy, mean intersection over union ( $IoU$), and dice coefficient. However, pixel accuracy doesn’t perform well in case of class imbalance in images because the dominant class will overlook other classes and result in unexpected outcomes. On the other hand, dice and IoU are proven to be a better choices for semantic segmentation in general [37], [38]. IoU and Dice evaluation matrices are similar and positively correlated since DICE is twice the amount of IoU as shown in equations 5 and 6 to measure the similarity between ground truth A and predicted segmentation B. In our study, we also used pixel accuracy (eq. 7), the F1 score (eq. 8), and MCC (Matthews Correlation Coefficient) (eq. 9 for evaluating the proposed approach [39], (5)–(9), as shown at the bottom of the page.

**Figure:** \begin{align*} IoU(A,B)=&\frac {|A \cap B|} {|A|\cup |B|} \tag{5}\\ DICE(A,B)=&\frac {2 |A \cap B|} {|A|\cup |B|} \tag{6}\\ Accuracy=&\frac {TP+TN}{TP+TN+FP+FN} \tag{7}\\ F1 Score=&\frac {2*Precision*Recall}{Precision+Recall} =\frac {2*TP}{2*TP+FP+FN} \tag{8}\\ MCC=&\frac {(TP * TN) - (FP * FN)}{\sqrt []{(TP + FP) * (TP + FN) * (TN + FP) * (TN + FN)}}\tag{9}\end{align*}

### B. Hardware Specification

The proposed framework ran on the server with the following hardware and software specifications: 2x 16-core Intel Xeon CPUs, 2x NVidia Titan 12GB GPUs, 128 GB RAM, 6 TB HDD storage, Ubuntu 18.04.5 LTS, Nvidia GPU driver v460.91, CUDA 11.2 + CuDNN 8.1, Torch v1.10.0, torchvision v0.11.1, and Spyder v4.2.5.

## IV. Results

**FIGURE 6.**
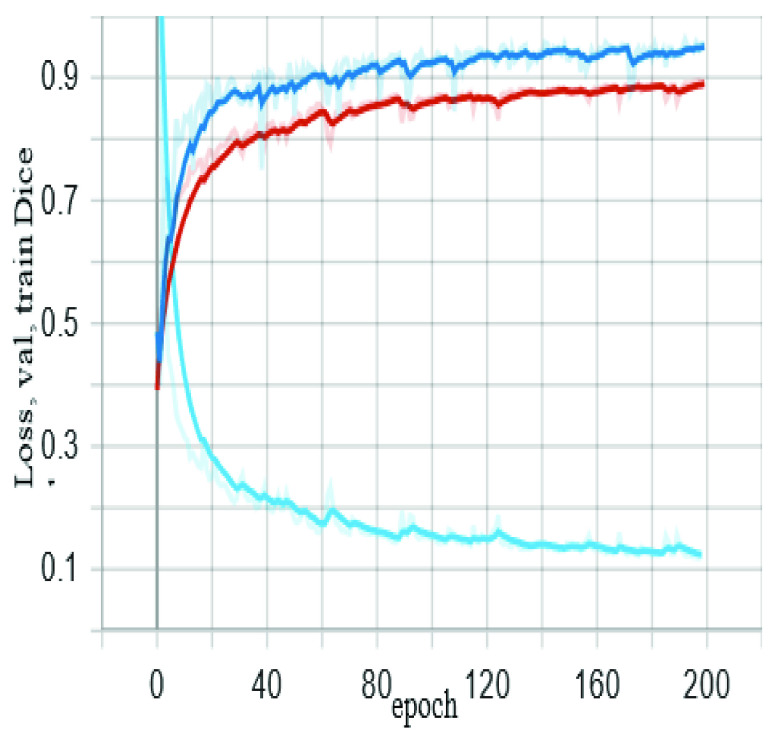
The proposed algorithm training and validation performance.

The training process on TCGA – LGG dataset for 200 epochs showed a validation dice of 0.96 for detecting and segmenting the brain tumor on MR images. Each epoch required 2-3 minutes using hardware specification in section 2.5. In data science applications, we usually divide the data into two parts - the training set, and the testing set. The training set is also split into two parts, the model training set and model validation set, the purpose of dividing the training set into two parts is to avoid overfitting during the training process, and to ensure that model evaluation (during training) is performed using an unseen set of data. Figure 6 shows the proposed algorithm training and validation performance (red and dark blue curves, respectively), and model loss is a light blue curve.

We compared the performance of the proposed framework with the well-known segmentation algorithm Unet. The testing was performed on 432 random MR images. The results show that Znet outperforms Unet in regards to dice, dice loss, pixel accuracy, F1 score, and MCC as shown in Table 4.

**TABLE 4:** Evaluation Metrics Comparison Between the Proposed Approach (Znet) and the Benchmark Algorithm Unet

	Unet	Znet (proposed)
Testing DICE	0.8544	0.9158
Testing DICE_loss	0.1452	0.0839
Pixels Accuracy	0.9922	0.9955
F1 Score	0.7878	0.8098
MCC	0.7839	0.8089
Trainable parameters	14,788,929	44,384,833

**FIGURE 7.**
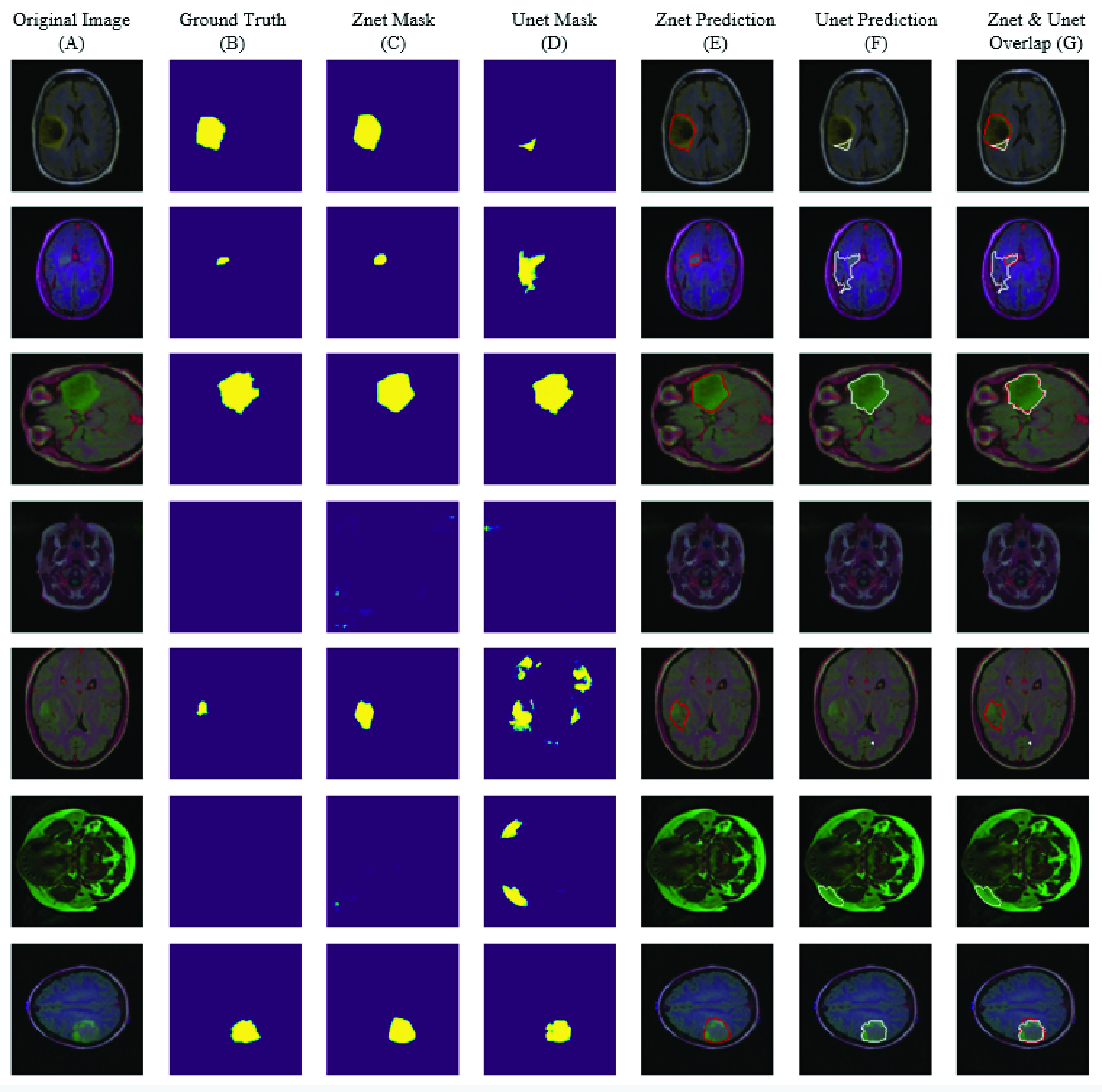
Visual results and comparison of MR images tumor segmentation using the proposed model (Znet) and the benchmark model Unet.

Figure 7 shows samples of auto segmentation for MR images using the proposed framework and the Unet model. Part (A) shows the original images before the segmentation process, (B) shows the ground truth for tumor location and shape in the original image, (C) indicates the produced mask (location and shape) for the tumor using Znet model, (D) displays the constructed mask for tumor using Unet model, (E) shows the auto segmentation of tumor using the Znet model, (F) is the corresponding segmentation using Unet, and (G) is the overlap between Znet and Unet. The analysis of the 432 testing dataset indicates that the Znet model can predict and segment the tumor in MR images with a dice value of about 0.92 and dice loss of 0.08.

## V. Discussion and Conclusion

In this work, we proposed a new approach for MR images segmentation based on the deep learning concept of convolutional network and data augmentation to utilize the available labeled images. The architecture relies on auto encoder-decoder, the concept of skip-connections, and residual neural networks, which requires combining the output of the previous layer with the next layer. Also features maps are required to map the dimensions between the input and the output of each layer. The benefit of skip-connections is to find alternative and further paths for the learning process and the gradient, which increase the probability of model convergence and avoid vanishing gradients dilemma [40]. The model was trained for 200 epochs using a server equipped with 2x NVidia Titan 12GB GPUs and showed a dice accuracy of 0.96 during training and about 0.92 for the testing dataset. Other measurements include pixel accuracy of 0.996, and 0.81for F1 score and MCC. For the benchmark Unet, the performance measures were 0.85 for dice, 0.992 for pixels accuracy, and 0.79 for F1 score and MCC. The evaluation metrics and the visualization of auto segmentation in the testing dataset (Figure 6) show the capability of the proposed approach for diagnosing and auto-segmentation of MR images of brain tumors. We can confirm that pixel accuracy is not a suitable evaluation measure for semantic segmentation in presence of class imbalance, just like in this case of MR images segmentation. Because the dominant class in ground truth images is the background. Also, high pixel accuracy can be misleading in some computer vision applications. On the other hand, some evaluation metrics, such as dice and IoU, provide more accurate assessment of the performance of alternative semantic segmentation techniques.

In the future, we will report about a prospective improvement and extension of the proposed Znet architecture to enable classification, extraction, parcellation, and prediction of the presence and extent of brain tumors using multichannel 3D MRI volumes. Predictive analytics and modeling of high-dimensional neuroimaging hyper-volumes is challenging for a number of reasons, e.g., complexities of representing higher-order data tensors, computational barriers, and the natural exponential increase of the state space of the optimization problem. Training supervised techniques such as Znet requires a large number of annotated ground truths (labels). Acquiring such large-scale testing and validation data is resource-intensive and time-consuming. We are also working on deep learning strategies to generate realistic high-dimensional and multimodal neuroimaging data along with ground truth labels. The transfer learning approach can be utilized along with the proposed Znet to optimize the training and minimize the costs of developing, validating, and embedding AI techniques in clinical practice settings.
